# The Effect of *Convolvulus arvensis* Dried Extract as a Potential Antioxidant in Food Models

**DOI:** 10.3390/antiox4010170

**Published:** 2015-03-10

**Authors:** Nurul Aini Mohd Azman, Maria Gabriela Gallego, Luis Juliá, Lluis Fajari, MaríaPilar Almajano

**Affiliations:** 1 Chemical Engineering Department, Technical University of Catalonia, Av. Diagonal 647, 08028 Barcelona, Spain; E-Mails: ainiazman@gmail.com (N.A.M.A.); maria.gabriela.gallego (M.G.G.); 2 Chemical and Natural Resources Engineering Faculty, University Malaysia Pahang, Lebuhraya Tun Razak, 26300 Pahang, Malaysia; 3 Química Biològica i Modelització Molecular, Institut de Química Avançada de Catalunya (CSIC), Jordi Girona 18-26, 08034 Barcelona, Spain; E-Mails: ljbmoh@cid.csic.es (L.J.); lluis.fajari@iqac.csic.es (L.F.)

**Keywords:** *Convolvulus arvensis*, lipid oxidation, active packaging film, antioxidant activity

## Abstract

In this study, the antioxidant activity of the *Convolvulus arvensis Linn* (CA) ethanol extract has been evaluated by different ways. The antioxidant activity of the extract assessed by 2,2′-azino-bis-3-ethylbenzothiazoline-6-sulphonic acid (ABTS) radical cation, the oxygen radical absorbance capacity (ORAC) and the ferric reducing antioxidant power (FRAP) was 1.62 mmol Trolox equivalents (TE)/g DW, 1.71 mmol TE/g DW and 2.11 mmol TE/g DW, respectively. CA ethanol extract exhibited scavenging activity against the methoxy radical initiated by the Fenton reaction and measured by Electron Paramagnetic Resonance (EPR). The antioxidant effects of lyophilised CA measured in beef patties containing 0.1% and 0.3% (w/w) CA stored in modified atmosphere packaging (MAP) (80% O_2_ and 20% CO_2_) was determined. A preliminary study of gelatine based film containing CA showed a strong antioxidant effect in preventing the degradation of lipid in muscle food. Thus, the present results indicate that CA extract can be used as a natural food antioxidant.

## 1. Introduction

Free radicals produced in the human body result from natural biochemical reactions and, together with external attacks due to stress, smoke and unbalanced diets, among other factors, could cause an imbalance between oxidants and antioxidants. For this reason, it is necessary to supplement the diet with antioxidant based food. This excess of radicals is associated with aging and many diseases such as heart problems, diabetes, neurodegenerative disorder and cancers. Previous studies indicate that the consumption of plant foods rich in antioxidants is beneficial for health and helps to prevent degenerative processes which contribute to many diseases [1,2,3]. Due to the increasing awareness of the benefits of consuming healthy food, many food companies are using antioxidants as an alternative approach, instead of using synthetic preservatives which at high doses may have toxic effects on the consumer.

Natural antioxidants are compounds, generally from plants, that are used as food additives with the aim of inhibiting oxidation of the product [4]. Thus, the use of natural antioxidants as preservatives to maintain quality and nutritional traits is increasingly widespread, mainly in food that contains high levels of lipids, such as meat products. Therefore, the incorporation of natural antioxidants such as herbs could be an economical strategy to develop healthier meat products. Moreover, they can improve technological properties, as well as increase the eco-efficiency [5] in the food industry. Besides formulation of food with a natural antioxidant strategy, active packaging is also gaining interest for its potential to provide food quality and safety benefits. The combination of natural preservatives and biodegradable plastic into one food packaging formulation is a promising approach to extending product shelf life [6].

Plants rich in polyphenol constituents possess antioxidant activity by free radical scavenging. For instance, green tea can inhibit lipid peroxidation and chelate transition metals, consequently helping to prevent degenerative diseases. If incorporated into an edible film, it could help to maintain the quality of food products [7].

*Convolvulus arvensis Linn* (CA) is an annual (or sometimes perennial climber), commonly found as a weed throughout Europe and Asia. This plant is being used for many purposes. The root and the resin are cholagogue, diuretic, laxative and purgative [8]. The flower is laxative, used as a tea infusion and also in treatment of wounds and fever, whereas the leaf can be helpful during the menstrual period [9]. Meanwhile, Meng *et al.* showed that the ubiquitous CA extract could be considered as a promising anti-cancer agent, with over 50% inhibition of tumor growth activity at non-toxic doses [10]. CA also provided an immunostimulant effect when tested on rabbits and turned out to have cytotoxic effects on human cancerous cells [11,12]. In a preliminary study, Thrakal *et al.* reported the antioxidant activity of CA extract using the DPPH method, nitric oxide scavenging activity and the reducing power assay [13]. Furthermore, the CA extract showed abundant traces of phenolic compounds including *p*-hydrobenzoic acid, syringic acid, vanillin, benzoic acid and ferulic acid [14]. This high content of phenolic compounds may allow it to serve as an antioxidant source for the food industry. However, the antioxidant activity of the CA extract towards lipid oxidation has not been fully determined yet. Thus, our goals were (1) to evaluate the antioxidant activity of CA using *in vitro* assays including FRAP, TEAC, ORAC and EPR scavenging activity and (2) to demonstrate the ability of CA extract to inhibit lipid deterioration in beef meat, by adding the dry extract directly in the patty composition or in the formulation with active packaging. One of the components in CA is an alkaloid, which is a compound that exhibits anti-cancer activity but may display toxic effects in the host at high doses. Therefore, the extraction of CA has been carried out according to the method described by Meng *et al.* [10] to reduce the presence of alkaloid in the extract before adding the lyophilized extract directly into the beef.

## 2. Experimental Section

### 2.1. Plant Material

Commercial dried CA was kindly supplied by Pàmies Hortícoles (Balaguer, Spain), a registered herbal company. All reagents and solvents used were of analytical grade and obtained from Panreac (Barcelona, Spain) and Sigma Aldrich (Gillingham, England).

### 2.2. Extraction of CA Extract

Dried roots of CA were finely ground using a standard kitchen food processor. Ground CA was extracted in three different ways: (1) with 50:50 (v/v) ethanol:water; (2) with 75:25 (v/v) ethanol:water and (3) with 90:10 (v/v) ethanol:water, always in the ratio 1:30 (w/v). The extractions were performed at 4 °C ± 1 °C for 24 h, in the dark with constant stirring. The extract solutions of CA were recovered by filtration using Whatman Filter paper, 0.45 μm (Whatman, GE healthcare, Wauwatosa, WI, USA). Part of the supernatant was taken for subsequent use to determine the antiradical capacity. The volume of the remaining supernatant was measured and the excess of ethanol was removed under vacuum using a rotary evaporator (Buchi Re111, Switzerland) and kept frozen at −80 °C for 24 h. All extracts were dried in a freeze dryer (Unicryo MC2L −60 °C, Germany) under vacuum at −60 °C for three days to remove moisture. Finally, lyophilised CA was weighed to determine the soluble solids concentration (g/L) as described by Zhang *et al*. [15]

### 2.3. Determination of the Total Phenolic Content (TPC)

The Folin-Ciocalteu method was used to determine the total phenolic content (TPC) as reported by Santas *et al.* [16].

### 2.4. Determination of Free Radical Scavenging Activity Assays

#### 2.4.1. *In-Vitro* Antioxidant Capacity Determination

Three different methods were used for the evaluation of the antioxidant activity of the extracts: 2,2′-azino-bis-(3-ethylbenzthiazoline)-6-sulphonic acid TEAC assay [17], Oxygen Radical Absorbance Capacity (ORAC) assay [18] and Ferric Reducing Antioxidant Power (FRAP) method [19]. Results were expressed as μM of Trolox equivalent (TE) per gram of dry weight of plant (DW).

#### 2.4.2. Electron Paramagnetic Resonance (EPR) Spectroscopy Radical Scavenging Assay

EPR radical scavenging activity was measured following the method described by Azman *et al.* [20]. The extraction was executed in MeOH in 1:10 (w/v) ratio and the soluble concentration of CA was determined according to the procedure above. The spin-trapping reaction mixture consisted of 100 μL of DMPO (35 mM); 50 μL of H_2_O_2_ (10 mM); 50 μL CA extract at different concentrations or 50 μL of ferulic acid used as reference (0–20 g/L) or 50 μL of pure MeOH used as a control; and, finally, 50 μL of FeSO_4_ (2 mM), added in this order. The final solutions (125 μL) were passed through a narrow (inside diameter = 2 mm) quartz tube and introduced into the cavity of the EPR spectrometer. The spectrum was recorded 10 min after the addition of the FeSO_4_ solution, when the radical adduct signal is greatest.

X-band EPR spectra were recorded with a Bruker EMX-Plus 10/12 spectrometer under the following conditions: microwave frequency, 9.8762 GHz; microwave power, 30.27 mW; center field, 3522.7 G; sweep width, 100 G; receiver gain, 5.02 × 10^4^; modulation frequency, 100 kHz; modulation amplitude, 1.86 G; time constant, 40.96 ms; conversion time, 203.0 ms.

### 2.5. Determination of Antioxidant Activity in Food Model

#### 2.5.1. Preparation of Beef Patties

The meat consisted of flank of beef provided by “Embutidos La Masia”, Barcelona. It was collected seven days after slaughter to allow it to mature and was kept at approximately −20 °C for further treatment. The extraction of CA was carried out according to the method used by Meng *et al.* to remove alkaloid compounds [10]. Fat and joint tissues were trimmed off lean meat (2000 g) and the meat was minced through 8 mm industrial plates. Then, the minced meat was divided into four batches and mixed with 1.5% of NaCl and either (i) control (no addition), (ii) 0.1% BHT, (iii) 0.1% lyophilised CA, (iv) 0.3% lyophilised CA. All batches were mixed vigorously for 2 min to attain an even distribution of additives throughout the meat. Each sample was moulded into smaller portions (about 20 g each), stuffed and packed with polystyrene B5-37 (Aerpack) trays and placed in BB4L bags (Cryovac) of low gas permeability (8–12 cm^3^·m^−2^ per 24 h). The air in the packaged trays was flushed with 80:20 (v/v) O_2_:CO_2_ by EAP20 mixture (Carburos Metalicos, Barcelona). Samples were stored in the dark at 4 °C ± 2 °C for 10 days and the samples were analysed for oxidation by thiobarbituric acid reactive substances (TBARS) method, % metmyoglobin, colour, pH and microbial quality. Every measurement was carried out in triplicate each day for 10 days (except for microbiological analysis which was done every three days).

#### 2.5.2. TBARS Assay

The TBARS method was used to measure the extent of lipid oxidation over the storage period as described by Grau *et al.* [21]. Samples (1 g) were weighed in a tube and mixed with 3 g/L aqueous EDTA. Then, the sample was immediately mixed with 5 mL of thiobarbituric acid reagent using an Ultra-Turrax (IKA, Germany); at 32,000 rpm speed, for 2 min. All procedures were carried out in the dark and all samples were kept in ice. The mixture was incubated at 97 ± 1 °C in hot water for 10 min and shaken for 1 min during the process to form a homogeneous mixture. The liquid sample was recovered by filtration (Whatman Filter paper, 0.45 μm), and then it was cooled for 10 min. The absorbance value of each sample was measured at 531 nm using a spectrophotometer. The TBARS value was calculated from a malonaldehyde (MDA) standard curve prepared with 1,1,3,3-tetraethoxypropane and analysed by linear regression. All results were reported in mg malonaldehyde per kg of sample (mg MDA/kg sample).

#### 2.5.3. Colour Measurement

Objective measurements of colour were performed using a CR 400 colorimeter (Minolta, Osaka, Japan). Each patty was cut and the colour of the slices was measured three times at each point. A portable colorimeter with the settings: pulsed xenon arc lamp, 0° viewing angle geometry and aperture size 8 mm, was used to measure meat colour in the CIELAB space (Lightness, L*; redness, a*; yellowness, b* (CIE, 1978). Before each series of measurements, the instrument was calibrated using a white ceramic tile.

#### 2.5.4. Percentage of Metmyoglobin

The metmyoglobin method was based on that developed by Xu *et al.* [22]. Five grams of beef patties were homogenized with 25 mL of ice-cold 0.04 M phosphate buffer (pH 6.8) for 15 s using a homogenizer (Ultra-Turrax, IKA, Germany), which was set at speed setting 2 (18,000 rpm). The homogenised patty was allowed to stand at 4 °C for 1 h and centrifuged at 4500 g for 20 min at 4 °C using a high-speed freezing centrifuge (GI-20G, Anke, Shanghai, China). The absorbance of the filtered supernatant was read at 572, 565, 545, and 525 nm with a spectrometer (Fluostar Omega, BMG Labtech, Germany). The percentage of metmyoglobin was determined using the formula: MetMb (%) = [−2.514 (A572/A525) + 0.777 (A565/A525) + 0.8 (A545/A525) + 1.098] × 100

#### 2.5.5. Development of Gelatin-Film with Antioxidant Coating

The fabrication of gelatin based film with antioxidant coating was adapted and characterized from Bodini *et al.* [23]. While the filmogenic solution was cooled after the solubilization of sorbitol, 0.75% (w/w) of CA extract / gelatin and 0.1% (w/w) BHT/gelatin were added.

### 2.6. Statistical Analysis

A one-way analysis of variance (ANOVA) was performed using Minitab 16 software program (Minitab Pty Ltd., Sydney, Australia) (α = 0.05). The results were presented as mean values (*n* ≥ 3).

## 3. Results and Discussion

### 3.1. Analysis of Total Polyphenols and Free Radical Activity Assays

On average, a higher weight of soluble solids was extracted from CA with 50% ethanol than with 75% and 90% of ethanol. The use of ethanol as extraction solvent is due to the fact that the solvent is recognized as a GRAS (Generally Recognized as Safe) component which can be safely used for applications in the food industry [24]. Ethanol also turned out to be effective in the extraction of flavonoids and their glycosides, catechols and tannins from raw plant materials. Generally, CA extracted with 50% ethanol showed higher phenolic content and antioxidant activity values in ORAC, FRAP and TEAC. Our results showed that the total phenolic content correlated with the antioxidant activity determined by the assays. Nevertheless, the values obtained in the ORAC assay were higher than the ones in the FRAP and TEAC assays, which also showed the extract scavenging activity against peroxy radicals (OOH^•^) generated in the assay. Total phenolic content reported for the plant extract with ethyl acetate turned out to be higher than our present results with 244 mg GAE/g DW [24]. The presence of compounds with antioxidant potential in the ethanol extract (Table 1) was revealed in the measurement of total antioxidant capacity in this study. In previous studies, the antioxidant activity of CA has been analyzed using the DPPH method, nitric oxide scavenging activity and reducing power assay applied to both methanol and ethyl acetate solvent extracts [13,25]. To the best of our knowledge, this is the first report of the antioxidant activity of CA extracts assessed using the TEAC, ORAC and FRAP methods.

**Table 1:** Soluble solids concentration, total phenolic content (TPC) and antioxidant activity of *Convolvulus arvensis Linn* (CA) extract.

Activity *Convolvulus arvensis*	Extraction Solvent		
	50:50 EtOH:H_2_O	75:25 EtOH:H_2_O	90:10 EtOH:H_2_O
Soluble concentration (g/L)	13.76 ± 0.05	13.61 ± 0.02	11.43 ± 0.05
Total phenolic content (g GAE/g DW)	13.0 ± 0.05	12.1 ± 0.03	9.9 ± 0.02
FRAP (mmol of TE/g DW)	1.62 ± 0.02	1.51 ± 0.06	0.98 ± 0.01
TEAC (mmol of TE/g DW)	1.71 ± 0.01	1.68 ± 0.01	1.41 ± 0.04
ORAC (mmol of TE/g DW)	2.11 ± 0.05	2.05 ± 0.05	1.71 ± 0.03

* Mean value *n* = 3. The standard deviation for each assay is less than 5%. Gallic Acid Equivalent (GAE), Trolox Equivalent (TE), Dry Weight (DW).

### 3.2. EPR Scavenging Radical Assay

**Figure 1:**
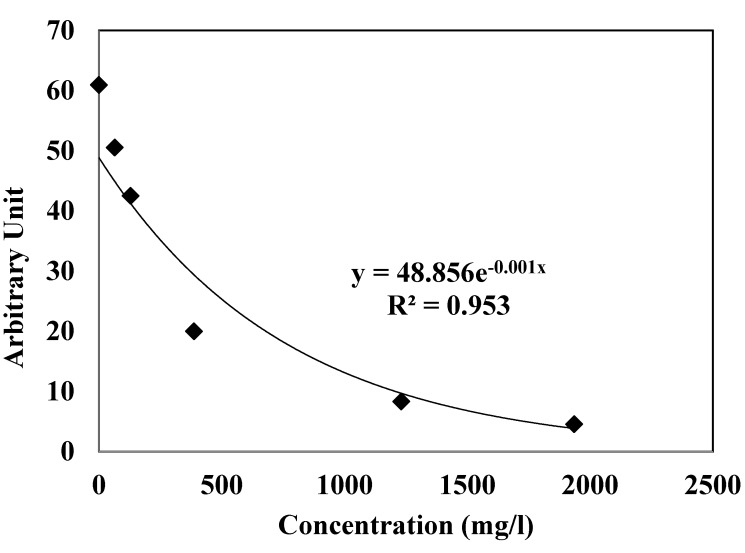
Antioxidant activity determined by the Electron paramagnetic resonance (EPR) spectrum of the radical adduct DMPO-OCH_3_ generated from a solution of H_2_O_2_ (2 mM) and FeSO_4_ (0.04 mM) with DMPO (14 mM) as spin trap in MeOH as solvent. The EPR signal decreases with the higher antioxidant activity.

The EPR radical scavenging method has been developed by Azman *et al.* to evaluate the concentration of free methoxy radicals (CH_3_O^•^) generated in the Fenton reaction with the CA extract [20]. Figure 1 shows the decreasing signal of EPR with the increase of CA extract concentration. The free radical scavenging activity of CA extracts was investigated against methoxy (CH_3_O^•^) radical by a competitive method in the presence of DMPO as spin trap, using EPR spectroscopy. CH_3_O^•^ was generated according to the Fenton procedure with a relatively short half-life that was identified by EPR because of its ability to form a stable nitroxide adduct with DMPO, DMPO-OCH_3_ (hyperfine splitting constants, a_N_ = 13.9 G and a_H_ = 8.3 G). This stable DMPO-OCH_3_ compound can be detected by the double integration value of the signal from EPR. The presence of CA extract at different concentrations may compete with the spin trap in the scavenging of methoxy radicals. Thus, the effect reduces the amount of radical adducts and, accordingly, reduces the intensity of the EPR signal. The best fitting with intensity of EPR signal was shown as an exponential function (Figure 1) that, if concentration values are in g/L, corresponds to Equation (1):

*y* = 48.856 e^−0.001 *x*^; *R*^2^ = 0.953 (1)

The graph indicates that the exponential value of the signal of the spectrum decreased as the amount of CA increased. This study confirmed that the scavenging activity of the *Convolvulus arvensis* extracts containing polyphenol constituents could be measured by the decrease of the intensity of the spectral bands of the adduct DMPO-OCH_3_ in the EPR spectrum with the amount of antioxidant.

### 3.3. Antioxidant Activity in Model Food

#### 3.3.1. Colour and % Metmyoglobin

Meat colour is one of the most important traits that reflect the meat freshness and quality for consumers. The colour parameters representing lightness (L*), redness (a*), and yellowness (b*) are shown in Table 2. Generally, the value of colour (L*, a* and b*) decreased as the storage time increased. Initial mean lightness (CIE L*) was 38.68 ± 0.87, and control sample showed the lowest value of L* at the end of 10 days storage. There are marginally differences in L* with all samples throughout storage times. The slight change of L* value in meat storage was addressed by few authors [26,27]. The decrease of L* value indicates that a darkening developed, which may be due to the Maillard reaction or the effect of moisture content, which influences lightness values [28,29].

**Table 2:** Effect of CA extract and BHT on instrumental colour value (L*, a*, b*) of beef patties during 10 days of refrigerated storage at 4 °C. (Mean ± SE).

Assay	Sample	Days of Storages					
		0	2	4	6	8	10
L*	Control	38.68 ± 0.87 ^a,1^	38.68 ± 1.50 ^a,1^	37.89 ± 0.32 ^b,3^	37.10 ± 1.23 ^b,2^	36.23 ± 0.45 ^c,2^	35.61 ± 2.22 ^d,1^
	0.1% BHT	38.68 ± 0.87 ^a,1^	39.06 ± 1.08 ^b,2^	38.25 ± 0.97 ^a,2^	38.43 ± 1.06 ^a,1^	37.09 ± 1.19 ^c,1^	36.18 ± 0.46 ^c,2^
	0.1% CA	38.68 ± 0.87 ^a,1^	38.60 ± 1.05 ^a,1^	39.26 ± 1.46 ^b,1^	38.63 ± 0.55 ^a,1^	37.11 ± 1.02 ^c,1^	37.06 ± 1.22 ^c,3^
	0.3 % CA	38.68 ± 0.87 ^a,1^	39.94 ± 0.71 ^b,2^	39.79 ± 1.23 ^b,1^	38.25 ± 1.40 ^a,1^	38.91 ± 1.47 ^a,3^	38.84 ± 1.13 ^a,4^
a*	Control	7.49 ± 0.27 ^a,1^	7.77 ± 0.29 ^a,1^	6.54 ± 0.33 ^b,1^	6.27 ± 0.16 ^b,2^	4.71 ± 0.02 ^c,1^	2.09 ± 0.01 ^d,1^
	0.1% BHT	7.49 ± 0.27 ^a,1^	8.18 ± 0.42 ^b,2^	9.28 ± 0.28 ^c,2^	7.05 ± 0.31 ^a,1^	6.36 ± 0.37 ^d,2^	2.87 ± 0.01 ^e,1^
	0.1% CA	7.49 ± 0.27 ^a,1^	7.61 ± 0.33 ^a,1^	5.57 ± 0.26 ^b,3^	6.25 ± 0.19 ^c,2^	6.60 ± 0.33 ^c,2^	3.31 ± 0.02 ^d,2^
	0.3 % CA	7.49 ± 0.27 ^a,1^	7.64 ± 0.21 ^a,1^	7.20 ± 0.47 ^a,4^	7.50 ± 0.20 ^a,1^	7.61 ± 0.37 ^a,3^	4.08 ± 0.01 ^b,3^
b*	Control	7.42 ± 0.32 ^a,1^	4.86 ± 0.01 ^b,1^	7.68 ± 0.36 ^a,1^	8.55 ± 0.19 ^c,1^	9.95 ± 0.21 ^d,1^	6.77 ± 0.02 ^e,1^
	0.1% BHT	7.42 ± 0.32 ^a,1^	6.68 ± 0.16 ^b,2^	8.40 ± 0.27 ^c,1^	8.39 ± 0.37 ^c,1^	8.38 ± 0.24 ^c,2^	6.10 ± 0.01 ^d,1^
	0.1% CA	7.42 ± 0.32 ^a,1^	8.00 ± 0.37 ^b,3^	8.19 ± 0.33 ^b,1^	5.17 ± 0.13 ^c,2^	7.49 ± 0.07 ^a,3^	4.35 ± 0.09 ^d,2^
	0.3 % CA	7.42 ± 0.32 ^a,1^	7.14 ± 0.49 ^a,4^	7.59 ± 0.29 ^a,2^	7.01 ± 0.21 ^a,3^	7.99 ± 0.27 ^a,3^	3.25 ± 0.01 ^b,3^

Control: 1.5% salt (w/w); 0.1% BHT: 1.5% salt with 0.1% BHT (w/w); 0.1% CA: 1.5% salt with 0.1% CA (w/w) 0.3% CA: 1.5% salt with 0.3% CA (w/w). ^a–d^: Means within a row with different letters are significantly different (*p* < 0.05). ^1–4^: For each attribute, means within a column with different number are significantly different (*p* < 0.05). Mean value *n* = 6 and the standard deviation for each assay is less than 5%.

A reduction of the a* value was experienced by all samples in 10 days’ storage (*p* < 0.05), indicating that a decrease in redness occurred in the meat. The 0.1% BHT displayed the highest value of a* during three days’ storage and declined gradually afterwards (*p* < 0.05). This finding was expected due to the role of BHT as a synthetic antioxidant which is used to retain colour and delay lipid oxidation in the meat [30]. The redness of 0.3% CA was maintained around a value of 7 during the eight days before the colour faded rapidly in 10 days’ storage (*p* > 0.05). At the end of storage, 0.3% CA showed the highest a* value followed by 0.1% CA (*p* < 0.05) and 0.1% BHT and control exhibited a low value with no significant difference between both samples (*p* > 0.05). Many features contributed to the red colour in the meat such as the influence of salt and oxygen composition that enhanced the red colour of beef patties [31,32]. The samples had an initial yellowness (b*) value of 7.42 ion that enhanced the red in both samples (eight days before *p* > 0.05). In general, no significant difference (*p* > 0.05) was observed in b* values in all samples throughout storage. The present findings seem to be consistent with other research which found that yellowness in meat patties is not influenced by storage time and packaging conditions [26,33].

The effect of CA extracts and BHT on relative MetMb percentage in beef patties are presented in Table 3. A significant correlation between MetMb (%) and the instrumental colour features was reported previously [22]. The MetMb percentage increased as the storage time increased throughout the 10 days’ refrigeration, whereas the control showed the highest MetMb compared to all samples. The treated groups of CA extract and BHT had lower (*p* < 0.05) proportions of MetMb compared to the control at the end of storage. The acceleration of colour deterioration and lipid oxidation depended on many causes, including storage time, type of packaging and test system. Free radicals produced by lipid oxidation in meat are susceptible to initiating the reaction of oxidizing oxymyoglobin (red colour) to metmyoglobin (brown colour) which results in the discolouration of meat during storage. Previous research has indicated a relationship between lipid oxidation and myoglobin oxidation or discolouration in meat products [22,34]. A sufficient amount of antioxidant in the sample can delay the formation of metmyoglobin. The scavenging ability of samples treated with antioxidant can reduce the oxidation of metmyoglobin acting as scavengers of hydroxyl radicals produced from oxidation of oxymyoglobin. The 0.3% of CA extract displayed the lowest metmyoglobin percentage compared to all samples, and the change of % metmyoglobin was inversely proportional to the value of redness (a*).

#### 3.3.2. TBARS Analysis in Beef Patties

**Figure 2:**
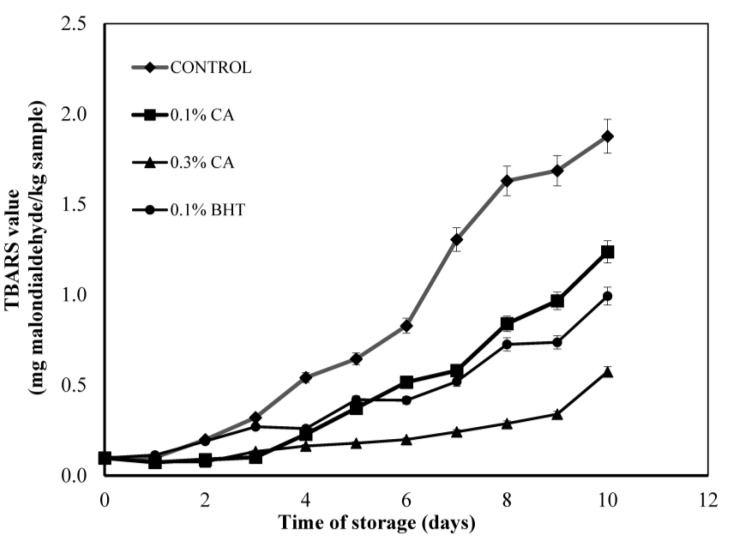
Changes in TBARS values (mg malondialdehyde/kg sample) of control and sample containing different concentrations (0.1% and 0.3% w/w) of CA extract in MAP atmosphere during 10 days storage at 4 ± 1 °C without light. Each sample was measured in triplicate and the average standard deviation for each sample was less than 5%.

In general, the levels of lipid oxidation in beef patties increased over time and the values followed the order: 0.3% CA < 0.1% BHT < 0.1% CA < Control (Figure 2). The presence of a controlled atmosphere with high oxygen packaging (MAP) resulted in higher TBARS values and increased the oxidation rate in muscle food [32,35]. No statistical difference was observed between 0.1% BHT and 0.1% CA on any of the storage days However, the TBARS values of both samples showed significant differences compared to those of the control samples (*p* < 0.05). From seven days onwards, the control reached the highest TBARS values of all samples, with values greater than 1.2 mg malonaldehyde/kg sample. The levels of lipid oxidation were the lowest in 0.3% CA in beef patties throughout storage and significantly lower than for all other samples. The oxidation rate of meat patties was more reduced for a higher concentration of CA extract, as shown by comparison of the rates for 0.1% and 0.3% addition. The 0.1% BHT was added for comparison with the natural antioxidant bearing in mind the FDA guidelines for using BHT is ≤200 ppm in meat products. The effect of CA extract on lipid oxidation in meat has never been reported. The active properties of CA reported by Hegab and Ghareib [14] have been attributed to various phenolic acids such as ferulic acid, cinnamic acid and *p*-coumaric acid. The antioxidant activity of phenolic compounds is closely related to the hydroxyl group linked to the aromatic ring which is capable of donating hydrogen atoms with electrons and neutralizing free radicals. This mechanism blocks further degradation by oxidation to form MDA, which can be measured by the TBARS method [36]. This study confirmed the potential of CA extract to inhibit lipid degradation in beef patties.

#### 3.3.3. TBARS Analysis in Meat under Active Packaging

**Figure 3:**
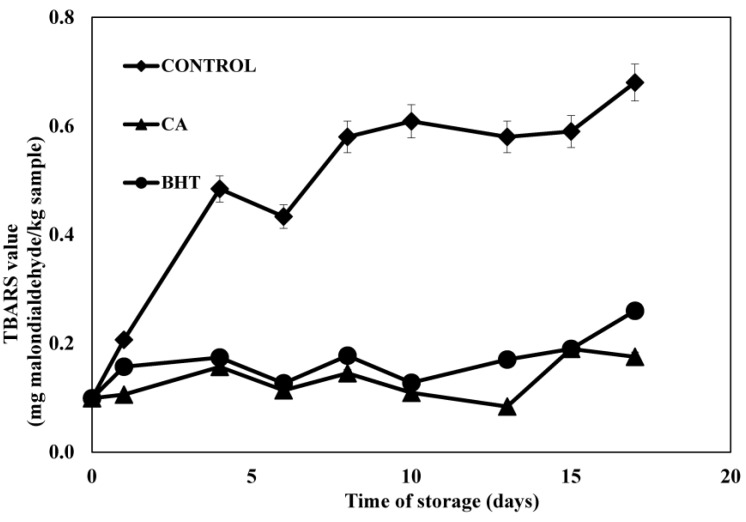
Changes in TBARS values (mg malondialdehyde/kg sample) of control and sample containing BHT and CA extract in MAP atmosphere during 17 days’ storage at 4 ± 1 °C without light. Each sample was measured in triplicate and the average standard deviation for each sample was less than 5%.

The TBARS index (Figure 3) revealed that the coating of beef patties with edible films enriched with antioxidants lowered the oxidation rate during 17 days’ storage. By comparison, the gelatin film without any added antioxidants did not display any protective effect. Lipid oxidation with respect to TBARS values of control, meat patties sample and those wrapped with CA and BHT incorporated film showed a significantly different TBARS value (*p* < 0.05) than the control sample. This result suggested that lipid oxidation in meat samples could be minimized by the use of a gelatin film containing CA probably due to the antioxidant activity of the CA extract. However, BHT and CA coated in gelatin film did not show any significant difference between the values for the different periods of storage.

Duthie *et al.* demonstrated the presence of phenolic acids measured using LC-MS in chicken patties mixed with vegetable powders including ferulic acid, *p*-hydrobenzoic acid, *p*-coumaric acid, caffeic acid and cinnamic acid [37]. In reviewing the literature, CA contained a great amount of phenolic compounds that may be responsible for its strong antioxidant activity in many assays. The constituents included *p*-hydroxybenzoic acid, syringic acid, vanillin, benzoic acid, ferulic acid found by Elzaawely and Tawata [25]. HPLC analysis done by Hegab and Ghareib showed traces of eight phenolic constituents including pyrogallic acid, protocatechuic acid, resorcinol, chologenic acid, caffeic acid, salicylic acid, *p*-coumaric acid and cinnamic acid [14]. These compounds lead to many pharmacological benefits to human health. Benzoic acid and its derivatives showed antimicrobial potential [38] while gallic acid and caffeic acid showed 50% inhibitory effects on cancer cell proliferation [39]. *p*-coumaric, ferulic acid and cinnamic acid and their derivatives bring many pharmacological benefits to humans including, anticancer and antioxidant effects [40,41]. Moreover, many constituents detected in the CA extract correlated significantly with antioxidant activity measured by ORAC and TEAC assays and have played an important role in the detoxification of endogenous compounds in humans [42].

**Table 3:** Effects of CA extract and BHT on metmyoglobin changes in beef patties during 10 days of refrigerated storage at 4 °C. (Mean ± SE).

Assay	Sample	Day of Storages					
		0	2	4	6	8	10
% Metmyoglobin	Control	23.38 ± 0.46 ^1^	29.19 ± 0.71 ^2^	37.56 ± 1.31 ^2^	47.84 ± 1.21 ^1^	53.9 ± 1.16 ^1^	60.03 ± 2.82 ^2^
	0.1% BHT	23.38 ± 0.46 ^1^	25.69 ± 1.04 ^4^	29.98 ± 0.81 ^1^	37.5 ± 1.85 ^2^	45.7 ± 1.53 ^2^	57.6 ± 1.24 ^1^
	0.1% CA	23.38 ± 0.46 ^1^	27.96 ± 0.33 ^1^	33.48 ± 0.79 ^3^	44.81 ± 1.29 ^3^	48.7 ± 1.67 ^3^	57.1 ± 1.18 ^1^
	0.3 % CA	23.38 ± 0.46 ^1^	28.91 ± 0.81 ^1,2^	30.71 ± 0.29 ^1^	33.37 ± 0.94 ^4^	40.1 ± 1.53 ^4^	50.78 ± 1.56 ^3^

Control: 1.5% salt (w/w); 0.1% BHT: 1.5% salt with 0.1% BHT (w/w); 0.1% CA: 1.5% salt with 0.1% CA (w/w) 0.3% CA: 1.5% salt with 0.3% CA (w/w). All samples values are significantly different throughout the storage time (*p* < 0.05) ^1–4^: Means within a column with different numbers are significantly different (*p* < 0.05). Mean value *n* = 6 and the standard deviation for each assay is less than 5%.

## 4. Conclusions

The CA extract showed an excellent antioxidant activity in 50% aqueous ethanol measured by FRAP, TEAC and ORAC assays. This is also the first time that the radical scavenging activity has been evaluated in a CA extract against methoxy radical generated in the Fenton Reaction assessed by EPR.

The CA extract also showed a protective effect against lipid degradation in the muscle food model. Lyophilised CA (0.1% and 0.3% w/w) can be applied as an antioxidant in meat patties. It showed inhibition of lipid oxidation in MAP. 0.3% of CA retained meat redness and browning colour measured by the metmyoglobin assay which was much better than the control (*p* < 0.05) during 10 days’ storage. A preliminary study of gelatin based film coated with CA showed there was a significant delay in the lipid degradation in beef (*p* < 0.05). Therefore, this study confirmed that CA could be used by the food industry as a source of antioxidants.
